# Supplementary material for: 1H-NMR Metabolomics Analysis of the Effect of Rubusoside on Serum Metabolites of Golden Hamsters on a High-Fat Diet
Source: Molecules. 2020 Mar 11;25(6):1274. doi: 10.3390/molecules25061274 (PMC7143983; doi:10.3390/molecules25061274)
Supplement: Supplementary file 1 [file molecules-25-01274-s001.pdf]

molecules

MDPI

Supplementary Materials

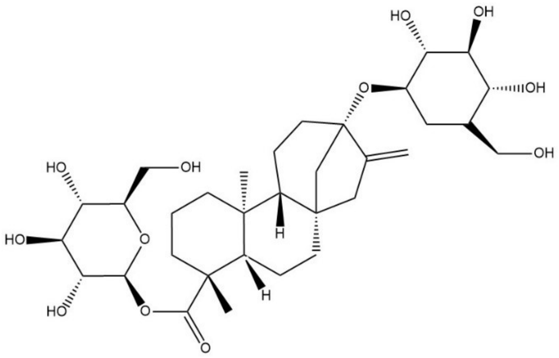

Figure S1. Molecular structure of rubusoside.

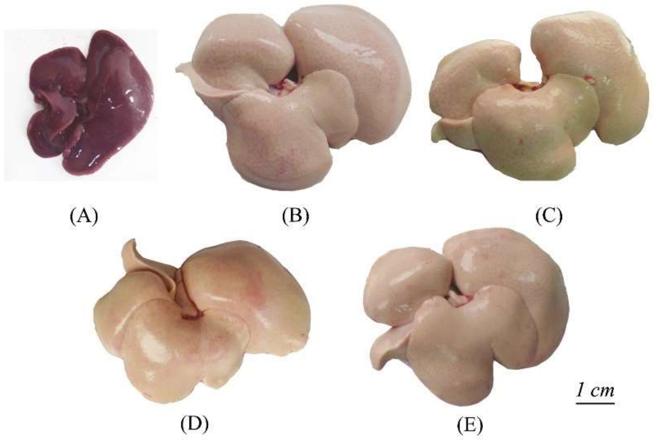

Figure S2. Images of livers from different experimental animal groups: (A) ND; (B) HFD; (C) HFD + SV; (D) HFD + PRbs; (E) HFD + Rbs.

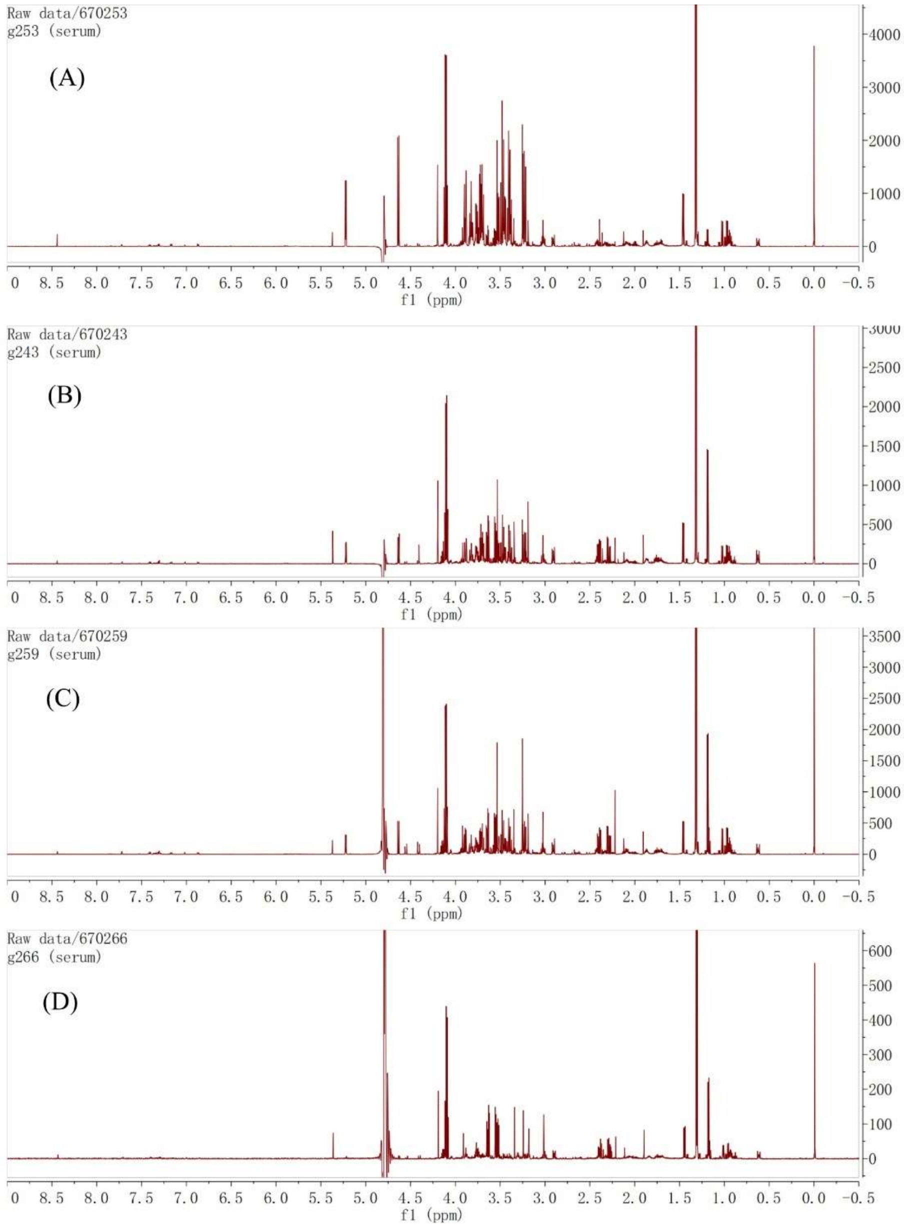

Figure S3. Representative serum ^{1}H NMR spectrum: (**A**) ND; (**B**) HFD; (**C**) HFD + PRbs; (**D**) HFD + Rbs.

Table S1. Metabolic pathway analysis of the serum of golden hamster (HFD vs ND).

Pathway name	Total Compounds	Hits	Raw p	-Log (p)	Holm adjust	FDR	Impact
Alanine, aspartate and glutamate metabolism	24	8	0.00	5.41	0.12	0.01	0.71
Synthesis and degradation of ketone bodies	6	3	0.00	9.14	0.00	0.00	0.70
Glycine, serine and threonine metabolism	48	9	0.00	19.01	0.00	0.00	0.44
Pyruvate metabolism	32	5	0.00	7.50	0.02	0.00	0.42
Arginine and proline metabolism	77	9	0.01	4.73	0.21	0.02	0.33
TCA cycle	20	6	0.01	4.35	0.28	0.02	0.31
Aminoacyl-tRNA biosynthesis	75	18	0.00	6.51	0.04	0.00	0.23
Methane metabolism	34	7	0.00	14.25	0.00	0.00	0.20
Glyoxylate and dicarboxylate metabolism	50	6	0.00	9.39	0.00	0.00	0.17
Butanoate metabolism	40	7	0.00	10.35	0.00	0.00	0.17
Lysine degradation	47	2	0.00	13.87	0.00	0.00	0.15
Histidine metabolism	44	4	0.00	7.34	0.02	0.00	0.14
D-Glutamine and D-glutamate metabolism	11	3	0.00	7.18	0.03	0.00	0.14
